# Two-dimensional ultrasound and two-dimensional shear wave elastography on femoral and saphenous neuropathy in patients with type 2 diabetes mellitus

**DOI:** 10.3389/fneur.2022.996199

**Published:** 2022-10-21

**Authors:** Yang Song, Ying Zhang, Yuhong Zhang, Bin Hu

**Affiliations:** ^1^Department of Diagnostic Ultrasound, The Second Hospital of Dalian Medical University, Dalian, China; ^2^Department of Diagnostic Ultrasound, Xinhua Affiliated Hospital of Dalian University, Dalian, China

**Keywords:** peripheral neuropathy, femoral nerve, saphenous nerve, diabetes, type 2, ultrasonography, elasticity imaging technique, diagnosis

## Abstract

**Objective:**

This study aims to examine the value of two-dimensional (2D) ultrasound and two-dimensional shear wave elastography (2D-SWE) in evaluating femoral nerve (FN) and saphenous nerve (SN) neuropathy in patients with type 2 diabetes mellitus (T2DM).

**Materials and methods:**

According to the diabetic peripheral neuropathy (DPN) diagnostic criteria, 60 patients with T2DM were enrolled and divided into 35 T2DM patients without DPN (non-DPN group) and 25 T2DM patients with DPN (DPN group). The control group consisted of another 15 healthy volunteers. The width, thickness, cross-sectional area (CSA), and perimeter of the FN and SN in the lower extremities were measured with 2D ultrasound. The average shear wave velocity (SWV) of the FN and SN was measured by 2D-SWE. Parameters of the left and right lower extremities were compared in each group, and the above parameters were compared among groups. The correlations between ultrasonographic and laboratory parameters were evaluated, and the independent influencing factors of SWV of the FN and SN were analyzed.

**Results:**

The width, thickness, CSA, perimeter, and SWV of FN and SN in the DPN group were greater than those in the non-DPN groups and control group (*P* < 0.05). The width, thickness, CSA, perimeter, and SWV of the FN and SN in the non-DPN group were greater than those in the control group (*P* < 0.05). The CSA of FN was positively correlated with FPG, HbA1c, and TG (*r* = 0.34–0.69, *P* < 0.01). The perimeter of FN was positively correlated with FPG, HbA1c, and TG (*r* = 0.37–0.68, *P* < 0.01). The perimeter of the FN was negatively correlated with IgF-1 (*r* = −0.31, *P* < 0.05). The CSA of the SN was positively correlated with FPG and TG (*r* = 0.26–0.42, *P* < 0.05). The perimeter of the SN was positively correlated with FPG and TG (*r* = 0.37–0.39, *P* < 0.01). The SWV of FN and SN were positively correlated with FPG and TG (*r* = 0.35–0.57, *P* < 0.01; *r* = 0.43–0.49, *P* < 0.01). FPG and TG were independent influencing factors of the SWV of the FN and SN (*P* < 0.05).

**Conclusion:**

2D ultrasound and 2D-SWE could be used to non-invasively, objectively, and accurately evaluate the abnormal changes of the FN and SN in patients with T2DM. It has important clinical significance for the early diagnosis of DPN and the curative effect evaluation.

## Introduction

Diabetic peripheral neuropathy (DPN) is one of the most common complications of diabetes mellitus and is a symmetrical polyneuropathy caused by metabolic and microvascular changes resulting from chronic hyperglycemia (1). Although DPN mainly involves distal nerves, it can be involved proximally, following a distal-proximal course (2). The most common early symptoms are induced by the involvement of tiny fibers and include pain and dysesthesia (unpleasant sensations of burning and tingling). The involvement of large fibers may cause numbness and loss of protective sensation (LOPS). LOPS indicates the presence of distal sensorimotor polyneuropathy and is a risk factor for diabetic foot ulceration (3). According to reports, approximately half of patients with diabetes have DPN, with approximately half of patients with DPN suffering from weakness and having a low quality of life due to pain (4). DPN pathogenesis is complex with an insidious onset and poor prognosis, and DPN is the major cause of disability in patients with diabetes mellitus.

Currently, two-dimensional (2D) ultrasound, due to its high resolution, has been used to examine peripheral nerves (5). In recent years, 2D-SWE has been a research hotspot in ultrasound. It uses acoustic radiation force impulse strain imaging to excite multiple tissue sites rapidly and continuously to form a nearly cylindrical shear wave cone, thereby obtaining tissue-specific hardness information (6, 7). Ultrasound elastography was first proposed by Ophir et al. in 1991 (8). It has played an active role in diagnosing thyroid, breast, liver, kidney, and some tumor diseases and has become a relatively well-established technology for clinical applications. It can quantitatively detect changes in mechanical information related to lesions in real time, thereby allowing early diagnosis of diseases. Although 2D-SWE has been gradually applied in the research and diagnosis of the musculoskeletal system (9, 10), there are few reports on the study of peripheral nerves using shear wave elastography. This study aims to explore the utility of 2D ultrasound and two-dimensional shear wave elastography (2D-SWE) for evaluating femoral nerve (FN) and saphenous nerve (SN) neuropathy in patients with type 2 diabetes mellitus (T2DM) and patients with DPN.

## Materials and methods

### Ethics and consent

This study was approved by the Ethics Committee of the Second Hospital of Dalian Medical University and was performed at the Department of Ultrasound [Approval Number: (2022) No. 039]. All patients and volunteers signed informed consent before the examination for statistical data analysis and publication of images.

### Participants

A total of 15 healthy volunteers and 60 patients diagnosed with T2DM in the Second Hospital of Dalian Medical University from November 2021 to April 2022 were enrolled. Inclusion criteria were as follows: all the patients with T2DM met the diagnostic criteria for T2DM established by WHO in 2019, including fasting plasma glucose ≥ 7.0 mmol/L or 2-h post-load plasma glucose ≥ 11.1 mmol/L (11). According to the clinical diagnostic criteria for DPN, including more than one symptom (foot tingling, numbness, ataxia, burning sensation, etc.) or distal symmetrical neuropathic pattern (abnormal knee or ankle reflexes, temperature, light touch, monofilament, or vibration sensation) (12), they were divided into two groups as follows: T2DM without DPN and T2DM with DPN after the neurological symptom score (NSS) and neurological sign score (NDS) used for assessment. The volunteers formed the control group. The T2DM without the DPN group was called the non-DPN group, and the T2DM with the DPN group was called the DPN group. Basic information such as age, height, gender, body mass index (BMI), and disease duration was collected. There was no significant difference in age, height, BMI, and gender among the groups. Exclusion criteria were as follows: secondary abnormal glucose metabolism caused by hereditary toxins and neurotoxic drugs; nerve abnormalities caused by nerve root compression, lumbar spinal stenosis, chronic inflammatory demyelinating neuropathy, and other cervical and lumbar diseases; lower extremity fractures, surgery, skin damage, and other patients who cannot complete ultrasound examination; other diseases that cause neuropathy such as hypothyroidism, vitamin B12 deficiency, HIV, multiple myeloma, etc. (13).

### Ultrasound examination

All US examinations were performed using a 4–15 MHz linear-array transducer (Resona8; Mindray, Guang Dong Provence, China) equipped with the 2D-SWE software. All subjects were lying supine, with semi-flexed knees and slightly external hip rotation. Both lower extremities were fully exposed and lay on the examination bed naturally. The bilateral FN and SN were scanned. The probe should be coated with an ultrasonographic coupling agent to isolate the interference of air evenly. At the same time, the strength applied to the transducer should be moderate during inspection to reduce the deformation effect on the nerve. The subjects were kept in the same environment during the measurement, and the room temperature was set at 25°C to reduce the effect of temperature on the elastic tissue of muscles and nerves.

#### 2D ultrasound examination

**Figure 1:**
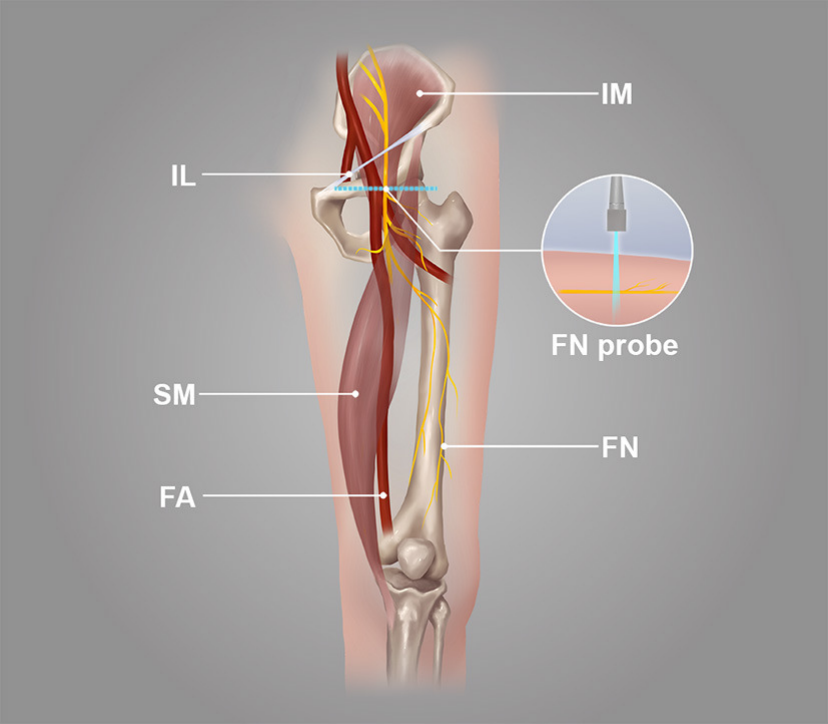
The position of the probe in the transverse section of the femoral nerve.

**Figure 2:**
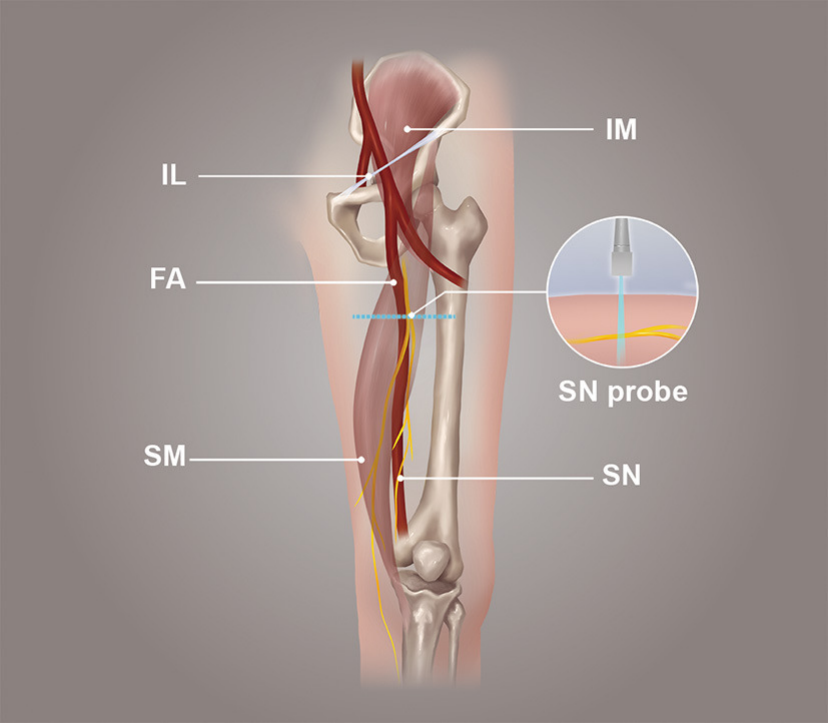
The position of the probe in the transverse section of the saphenous nerve.

**Figure 3:**
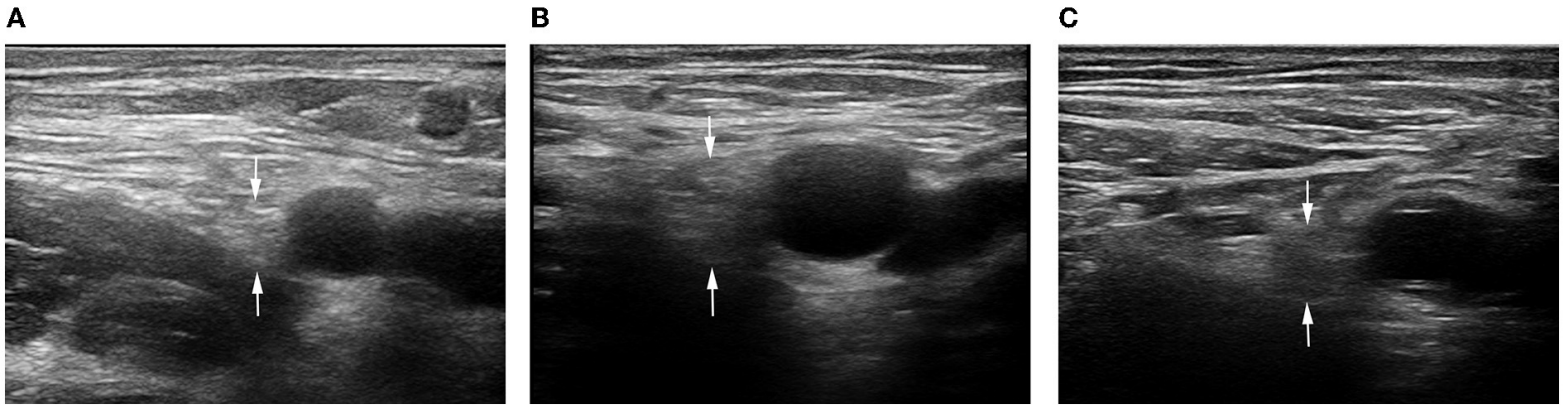
2D ultrasound image of the FN. **(A)** Control subject; **(B)** patient with T2DM, non-DPN group; **(C)** patient with T2DM, DPN group.

The probe was parallel to the inguinal ligament and placed approximately 5 cm below the inguinal ligament, on the medial side of the iliacus muscle, and lateral to the femoral artery (Figure 1). We considered this location a measurement area of the FN, to avoid the area where the nerve gives off its branches, and localized the nerve (14). Next, the probe was placed in the middle third of the thigh, below the sartorius muscle, anteromedial to the femoral artery (Figure 2). We decided on this location as the measurement location of the SN and searched for the SN (15). The normal FN and SN appeared as “honeycomb” triangular hyperechoic structures. At the above measurement sites, we obtained a short-axis image, freezing the image when it was clear and stable. Finally, the width and thickness of the FN and SN were measured, and the cross-sectional area (CSA) and perimeter were obtained by tracing from outside the hyperechogenic border of the nerve (Figure 3).

#### 2D-SWE ultrasound examination

**Figure 4:**
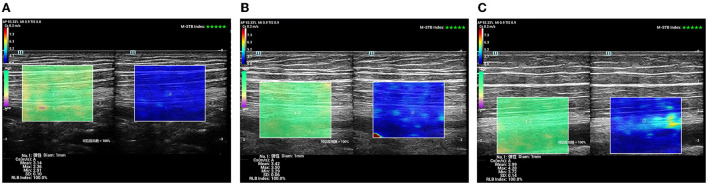
SWE sonogram of the SN. **(A)** Control subject; **(B)** patient with T2DM, non-DPN group; **(C)** patient with T2DM, DPN group.

It was a longitudinal or long-axis image. The SWE software started up while the image was clear and stable. When the image confidence was greater than 95%, we paused for 5 s to obtain a stable image and freeze it. The color map's shear wave velocity (SWV) range was set to 0–8.2 m/s, and the region of interest (ROI) was placed inside the epineurium of the FN and SN with a diameter of 1 mm. Then the system automatically calculated the mean shear wave velocity (SWV) value of the FN and SN in the region. A higher SWV in the ROI indicated a harder tissue. The blue color in the ROI indicated a softer average hardness, the yellow color indicated a moderate average hardness, and the red color indicated a harder average hardness (Figure 4).

### Laboratory examination

Fasting plasma glucose (FPG), hemoglobin A1C (HbA1c), insulin-like growth factor-1 (IgF-1), total cholesterol (TC), triglycerides (TG), and low-density lipoprotein cholesterol (LDL-C) examination results were recorded.

### Statistical analyses

GraphPad Prism version 9.0.0 for Windows (GraphPad Software, San Diego, California, USA) statistical software was applied for data processing and analysis. The χ^2^ test compared gender differences between groups, and continuous variables were expressed as means ± standard deviations. A one-way variance test (ANOVA) analysis was used to compare three groups. The Tukey test was used for multiple comparisons among groups, and the independent samples *t*-test was used for comparisons between two groups. Paired *t*-test was used to compare the left and right nerves. Pearson correlation analysis was used to evaluate the correlation between ultrasonographic and laboratory parameters. With laboratory parameters as independent variables and the SWV of the FN and SN as dependent variables, a multiple linear regression analysis was carried out to analyze the independent factors affecting the SWV of the FN and SN. *P* < 0.05 value was considered statistically significant.

## Results

### Study demographics

There were no significant differences in age, height, BMI, and gender among the three groups (*P* > 0.05). There were significant differences in disease duration, HbA1c, FPG, and TG between the DPN and non-DPN groups (*P* < 0.05), and the parameters were higher in the DPN group. However, there was no significant difference in IgF-1, TC, and LDL-C between the DPN and non-DPN groups (*P* > 0.05). The baseline demographic data of the control, DPN, and non-DPN groups are shown in Table 1.

**Table 1:** Characteristics of study participants.

	**Control group (*n =* 15)**	**Non-DPN group (*n =* 35)**	**DPN group (*n =* 25)**	***P* Value**
Age (years)	50.80 ± 8.85	53.03 ± 9.20	56.48 ± 10.60	0.17
Height (m)	167.50 ± 8.24	167.50 ± 7.56	167.50 ± 7.01	0.99
Sex (female)	7 (46.7%)	17 (48.6%)	12 (48%)	0.99
Body mass index (kg/m^2^)	24.73 ± 2.22	25.70 ± 4.79	24.53 ± 2.56	0.45
Disease duration (years)	NA	6.14 ± 4.53	16.16 ± 7.46^a^	< 0.01
HbA1c (%)	NA	8.16 ± 1.48	9.30 ± 2.85^a^	0.04
FPG (mmol/L)	NA	8.52 ± 3.07	16.81 ± 1.19^a^	< 0.01
IgF-1 (mmol/L)	NA	167.10 ± 42.35	151.30 ± 17.86	0.08
TG (mmol/L)	NA	0.75 ± 0.33	1.93 ± 1.64^a^	< 0.01
TC (mmol/L)	NA	5.06 ± 1.58	5.28 ± 1.39	0.58
LDL-C (mmol/L)	NA	3.36 ± 1.66	3.20 ± 0.98	0.67

Values are presented as mean ± SD or as a proportion. NA, not applicable.

^a^Compared with the non-DPN group, *P* < 0.05. *P* < 0.05 value was considered statistically significant.

HbA1c, hemoglobin A1C; FPG, fasting plasma glucose; IgF-1, insulin-like growth factor-1; TG, triglycerides; TC, total cholesterol; LDL-C, low-density lipoprotein cholesterol.

### Comparison of 2D ultrasound measurements

The differences in the width, thickness, CSA, and perimeter of the FN and SN among the three groups were statistically significant (*P* < 0.05). The width, thickness, CSA, and perimeter of the FN and SN in the DPN group were greater than those in the control and non-DPN groups (*P* < 0.05). The width, thickness, CSA, and perimeter of the FN and SN in the non-DPN group were greater than those in the control group (*P* < 0.05), as revealed in Table 2. There was no significant difference in CSA between the left and right FN and SN among the three groups (*P* > 0.05).

**Table 2:** Comparison of ultrasound parameters of the femoral nerve and saphenous nerve among subjects in three groups.

	**Control group (*n =* 30)**	**Non-DPN group (*n =* 70)**	**DPN group (*n =* 50)**	***P* Value**
FN	
Width (cm)	0.77 ± 0.10	0.90 ± 0.06^a^	1.04 ± 0.05^a, b^	< 0.01
Thickness (cm)	0.61 ± 0.07	0.70 ± 0.07^a^	0.83 ± 0.06^a, b^	< 0.01
CSA (cm^2^)	0.42 ± 0.11	0.57 ± 0.08^a^	0.76 ± 0.07^a, b^	< 0.01
Perimeter (cm)	2.37 ± 0.34	2.83 ± 0.25^a^	3.28 ± 0.20^a, b^	< 0.01
SWV (m/s)	3.14 ± 0.15	3.80 ± 0.26^a^	4.35 ± 0.42^a, b^	< 0.01
SN	
Width (cm)	0.32 ± 0.04	0.40 ± 0.06^a^	0.46 ± 0.04^a, b^	< 0.01
Thickness (cm)	0.24 ± 0.04	0.32 ± 0.06^a^	0.39 ± 0.05^a, b^	< 0.01
CSA (cm^2^)	0.08 ± 0.02	0.13 ± 0.04^a^	0.18 ± 0.04^a, b^	< 0.01
Perimeter (cm)	1.01 ± 0.12	1.35 ± 0.18^a^	1.54 ± 0.17^a, b^	< 0.01
SWV (m/s)	2.92 ± 0.15	3.52 ± 0.24^a^	3.75 ± 0.38^a, b^	< 0.01

^a^Compared with the control group, *P* < 0.01; ^b^compared with the non-DPN group, *P* < 0.01.

FN, femoral nerve; SN, saphenous nerve; CSA, cross-sectional area; SWV, shear wave velocity.

### Comparison of 2D-SWE ultrasound measurements

**Figure 5:**
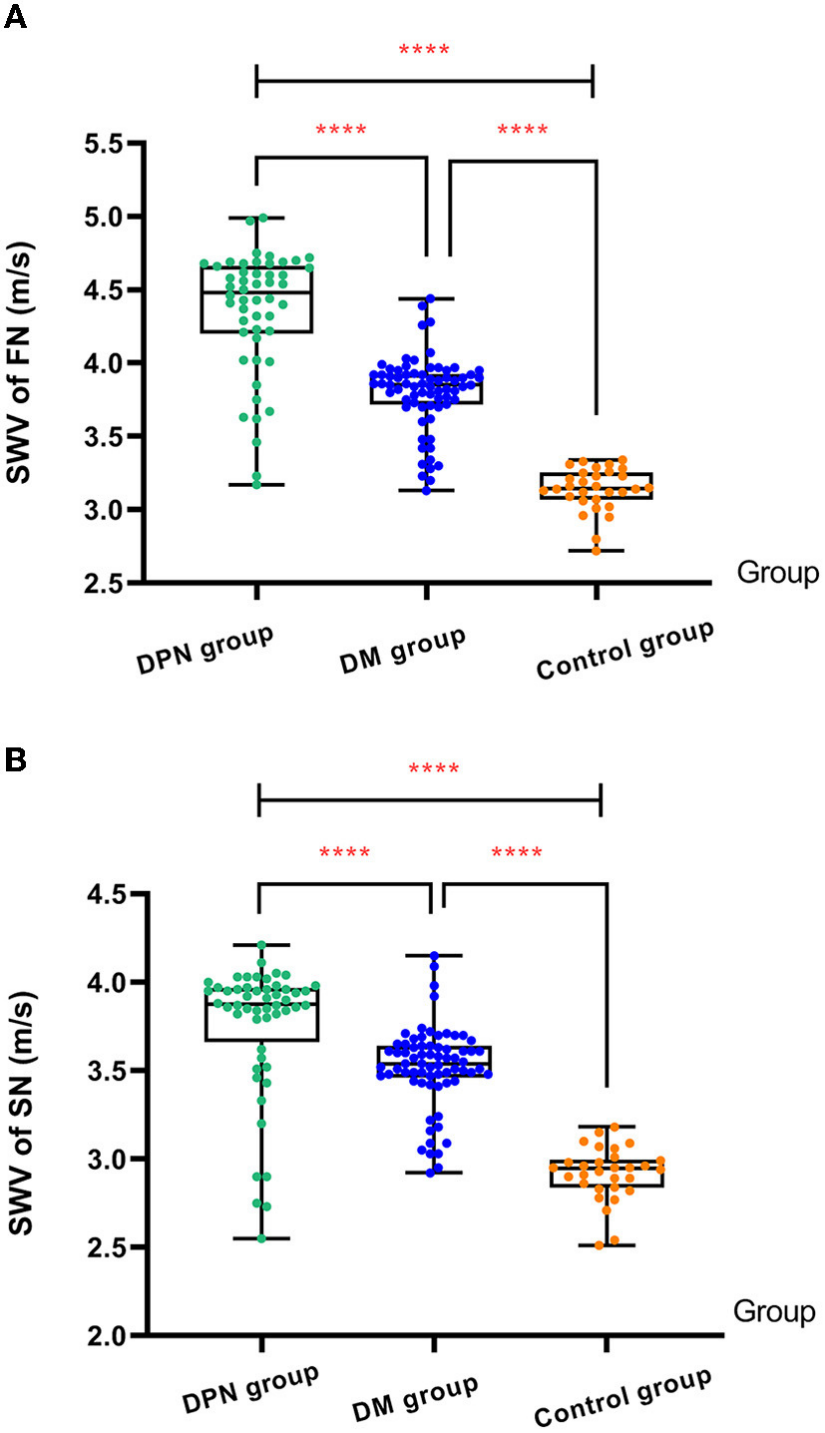
**(A)** Comparison of SWV of FN among three groups. **(B)** Comparison of SWV of SN among three groups. The ****symbol indicates the significant difference between groups.

The overall differences in SWV of the FN and SN among the three groups were statistically significant (*P* < 0.05). The SWV of the FN and SN in the DPN group was higher than that in the control and non-DPN groups (*P* < 0.05). The SWV of the FN and SN in the non-DPN group was higher than that in the control group (*P* < 0.05) (Figure 5). No significant difference in the SWV of the left and right FN and SN was found among the three groups (*P* > 0.05), as illustrated in Table 3.

**Table 3:** Comparison of CSA and SWV of the left and right FN and SN among three groups.

		**Control group (*n =* 15)**	**Non-DPN group (*n =* 35)**	**DPN group (*n =* 25)**
CSA	Left FN	0.42 ± 0.11	0.56 ± 0.09	0.76 ± 0.07
	Right FN	0.41 ± 0.11	0.57 ± 0.07	0.76 ± 0.07
	*P* Value	0.25	0.60	0.07
	Left SN	0.08 ± 0.02	0.13 ± 0.04	0.18 ± 0.04
	Right SN	0.08 ± 0.02	0.13 ± 0.04	0.18 ± 0.04
	*P* Value	0.77	0.94	0.45
SWV	Left FN	3.13 ± 0.16	3.79 ± 0.27	4.33 ± 0.42
	Right FN	3.15 ± 0.14	3.81 ± 0.25	4.37 ± 0.43
	*P* Value	0.10	0.36	0.16
	Left SN	2.91 ± 0.16	3.52 ± 0.24	3.71 ± 0.43
	Right SN	2.92 ± 0.15	3.52 ± 0.23	3.78 ± 0.35
	*P* Value	0.07	0.69	0.07

FN, femoral nerve; SN, saphenous nerve; CSA, cross-sectional area; SWV, shear wave velocity.

### Correlation analysis

The CSA of FN was positively correlated with FPG (*r* = 0.69, *P* < 0.01), HbA1c (*r* = 0.34, *P* < 0.01), and TG (*r* = 0.44, *P* < 0.01). The perimeter of FN was positively correlated with FPG (*r* = 0.68, *P* < 0.01), HbA1c (*r* = 0.37, *P* < 0.01), and TG (*r* = 0.41, *P* < 0.01). However, it was negatively correlated with IgF-1 (*r* = −0.31, *P* < 0.05). The CSA of the SN was positively correlated with FPG (*r* = 0.42, *P* < 0.01) and TG (*r* = 0.26, *P* < 0.05). The perimeter of the SN was positively correlated with FPG (*r* = 0.39, *P* < 0.01) and TG (*r* = 0.37, *P* < 0.01).The SWV of FN and SN were positively correlated with FPG (*r* = 0.57, *P* < 0.01; *r* = 0.37, *P* < 0.01) and TG (*r* = 0.49, *P* < 0.01; *r* = 0.43, *P* < 0.01).

### Linear regression analysis

Regarding FN and SN, the regression analysis showed that FPG and TG were independent influencing factors of the SWV (*P* < 0.05), as summarized in Table 4.

**Table 4:** Linear regression analysis of the SWV of FN and SN.

**Independent influencing factor**	**FN**			**SN**		
	**R^**2**^**	***P***	**95%CI**	**R^**2**^**	***P***	**95%CI**
FPG	0.33	< 0.01	3.95–8.70	0.13	< 0.01	1.86–9.37
TG	0.24	< 0.01	0.72–2.02	0.18	< 0.01	0.75–2.61
HbA1c	0.01	0.44	−0.81–1.84	0.00	0.82	−1.65–2.07
IgF-1	0.00	0.93	−22.03–20.24	0.01	0.41	−17.23–41.33
TC	0.01	0.48	−0.58–1.22	0.01	0.51	−0.84–1.68
LDL-C	0.00	0.66	−0.66–1.04	0.01	0.55	−0.82–1.54

FPG, fasting plasma glucose; TG, triglycerides; HbA1c, hemoglobin A1C; IgF-1, insulin-like growth factor-1; TC, total cholesterol; LDL-C, low-density lipoprotein cholesterol.

## Discussion

DPN is a chronic neurological disease caused by diabetes mellitus. The mechanisms of DPN are not fully understood but are currently believed to involve downstream injurious pathways associated with hyperglycemia, dyslipidemia, and microvascular disease leading to neuronal inflammation, oxidative stress, mitochondrial dysfunction, and ultimately cell death (1). Clinical diagnosis is mainly based on the patients' symptoms and signs combined with neurophysiological results (16, 17). Reliable neurophysiological results are related to filtering setting, limb temperature, and recording location. Moreover, it is not suitable as a routine screening tool as it is time-consuming and often brings discomfort to the participants. In addition, some early reports have depicted that patients with diabetes mellitus in the early stage are more likely to have neuropathy restricted to small nerves. However, neurophysiology is more sensitive to detecting changes in large nerve fibers (18–20), and its low detection rate of small fiber neuropathy often leads to missed diagnosis (21). Studies have demonstrated that ultrasonography can detect subclinical involvement of peripheral nerves, but it is negative in neurophysiological examination (18). As a non-invasive evaluation technology based on ultrasound, shear wave elastography has attracted more and more attention from doctors and researchers. Łukasz Paluch et al. applied 2D-SWE technology to the study of ulnar nerve entrapment. The results showed that the stiffness of the ulnar nerve in the cubital tunnel of ulnar neuropathy at the elbow patients was significantly greater than that of healthy controls (22). Andrade et al. used shear wave elastography to evaluate different effects of body position on sciatic nerve SWV. The results displayed that the sciatic nerve stiffness increased when the ankle joint was dorsiflexed. However, when the knee was flexed at 90°, its stiffness did not change significantly (23). It demonstrates that shear wave elastography can non-invasively assess the stiffness of the sciatic nerve during passive motion. However, to the best of our knowledge, the potential application of 2D-SWE to detect FN and SN in patients with T2DM has not been investigated so far.

In this study, 2D ultrasound and 2D-SWE technology were used to evaluate the FN and SN in patients with T2DM. The results showed that the width, thickness, CSA, and perimeter of the FN and SN in the DPN group were greater than in the non-DPN and control groups. Furthermore, the width, thickness, CSA, and perimeter of the FN and SN in the non-DPN group were larger than those in the control group. These results indicate that the degree of neuronal edema increases with the involvement of diabetes mellitus peripheral nerves. A large amount of glucose is converted into hypertonic sorbitol and fructose as the polyol pathway is activated during the progression of DPN. This pathway also leads to oxidative stress and dysfunction of Na+/K+ ATPase activity, further leading to water and sodium retention in nerve cells, cell edema, increased volume, and structural changes, resulting in increased CSA ultimately (1). There are few previous studies on the application of 2D ultrasound to the FN and SN in patients with diabetes mellitus and DPN. However, K. Singh et al. conducted a systematic study of the tibial nerve and found that the 2D ultrasound parameters of the tibial nerve in patients with DPN were greater than those in patients with non-DPN and those in patients with non-DPN were greater than those in healthy controls (24).

This study demonstrated that the SWV of FN and SN in the DPN group was higher than in the non-DPN and control groups. Furthermore, the SWV of FN and SN in the non-DPN group was higher than in the control group. These results suggest that nerve stiffness gradually increases with the involvement of peripheral nerves in diabetes mellitus. In long-term glucose metabolism disorders, DPN can lead to oxidative stress, mitochondrial dysfunction, and inflammation-mediated and immune-mediated neurotoxicity, and these pathways can further damage Schwann cells. Schwann cells are essential for the survival of peripheral nerves as they provide energy and protection for neurons. Concurrently, DPN can lead to endoneurial and exoneurial microangiopathy and endothelial dysfunction, which can further result in impaired nerve blood flow and endoneurial hypoxia. The above pathophysiological changes eventually lead to myelin disruption, demyelination, axonal conductance abnormalities, impaired neuronal regeneration with fibrotic nerve response, and increased stiffness (1). In this study, the 2D-SWE measurement parameter selected the average SWV value instead of the more commonly used Young's modulus value because previous studies by scholars have shown that in nerves, muscles, and other tissues with obvious anisotropy, the SWV to evaluate the hardness of the medium is more accurate than Young's modulus value (7). A certain degree of DPN changes has already appeared in patients with T2DM during diagnosis or in the pre-diabetic state. At this time, there is no obvious abnormality in the 2D ultrasonography of peripheral nerves, but abnormal changes in metabolism and stiffness have been observed (1, 25). 2D-SWE can obtain tissue stiffness information by measuring SWV, which indirectly reflects the changes in neural structure and prompts the occurrence of early lesions. Some scholars have used 2D-SWE technology to study the tibial nerve of patients with DPN, and the results showed that the stiffness of the tibial nerve in patients with DPN was significantly greater than that in non-DPN patients with healthy controls (26). However, there is no research on the application of 2D-SWE on FN and SN of patients with DPN.

This study also showed that the CSA of FN was positively correlated with FPG, HbA1c, and TG. The FN perimeter was positively correlated with FPG, HbA1c, and TG, while the FN perimeter was negatively correlated with IgF-1. The CSA of SN was positively correlated with FPG and TG. The perimeter of the SN was positively correlated with FPG and TG. The SWV of FN and SN was positively correlated with FPG and TG. FPG and TG were independent influencing factors of the SWV of FN and SN. The results showed that the increase in FPG, HbA1c, and TG and the decrease in IgF-1 were closely related to the occurrence and development of DPN. Studies have shown that hyperglycemia may be the most important factor in DPN. Proteins, lipids, and nucleic acids can undergo irreversible non-enzymatic reactions under hyperglycemic conditions, resulting in the formation of advanced glycation end products (AGEs) and the interaction of AGEs with their receptors (RAGE), ultimately resulting in vascular dysfunction and nerve conduction deficits. At the same time, previous studies have shown that a 1% increase in HbA1c results in an estimated 10% higher prevalence of DPN. Elevated serum levels of TG were related to a decline in myelinated fiber density and impaired neuronal mitochondrial trafficking and bioenergetic function, finally leading to neuronal cell death. The reduction in IgF-1 can lead to impaired signal transduction, dysregulation of neuronal nutritional support, reduced regeneration efficiency and accelerated DPN progression (1). Therefore, ultra-sonographic parameters combined with laboratory parameters are also of great significance for the diagnosis of DPN.

This study has some limitations. First, no neurophysiological data from FN and SN were obtained, and no gold standard result was available to compare. Second, the ligament and pulsation of the femoral artery adjacent to FN may affect the accuracy of the 2D-SWE measurement of FN. Finally, the branches of SN after entering the adductor canal are too small and with individual differences in the position, which cannot be displayed for measurement.

In conclusion, 2D ultrasound and 2D-SWE technology can non-invasively, objectively, and accurately evaluate the abnormal changes in FN and SN in patients with T2DM and DPN. 2D ultrasound and 2D-SWE technology may become routine diagnostic tools for the clinical detection of lower extremity peripheral neuropathy, which has significant clinical implications for the early diagnosis and treatment of DPN.

## Data availability statement

The original contributions presented in the study are included in the article/Supplementary material, further inquiries can be directed to the corresponding author/s.

## Ethics statement

The studies involving human participants were reviewed and approved by the Ethics Committee of the Second Hospital of Dalian Medical University. The patients/participants provided their written informed consent to participate in this study.

## Author contributions

YS and YiZ: writing-original draft preparation, formal analysis, visualization, methodology, and investigation. BH and YuZ: conceptualization, methodology, writing-review, and editing. YS and YuZ: resources and data curation. All authors have read and agreed to the published version of the manuscript.

## Conflict of interest

The authors declare that the research was conducted in the absence of any commercial or financial relationships that could be construed as a potential conflict of interest.

## Publisher's note

All claims expressed in this article are solely those of the authors and do not necessarily represent those of their affiliated organizations, or those of the publisher, the editors and the reviewers. Any product that may be evaluated in this article, or claim that may be made by its manufacturer, is not guaranteed or endorsed by the publisher.
